# Supplementary material for: IP6-assisted CSN-COP1 competition regulates a CRL4-ETV5 proteolytic checkpoint to safeguard glucose-induced insulin secretion
Source: Nat Commun. 2021 Apr 28;12:2461. doi: 10.1038/s41467-021-22941-3 (PMC8080631; doi:10.1038/s41467-021-22941-3)
Supplement: Supplementary file 1 — Supplementary Information [file 41467_2021_22941_MOESM1_ESM.pdf]

# Supplementary Information

## $IP_6$-assisted CSN-COP1 competition regulates a CRL4-ETV5 proteolytic checkpoint to safeguard glucose-induced insulin secretion against hyperinsulinemia

**Lin et al.**

## Supplementary Figure 1

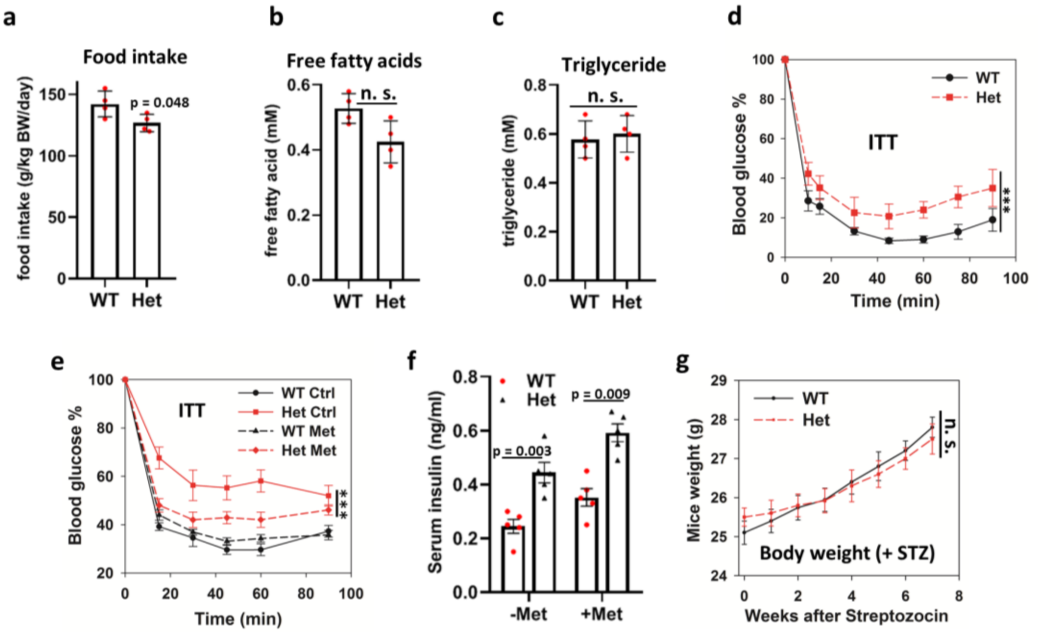


**Supplementary Fig. 1. Enhanced insulin secretion and congenital hyperinsulinemia in *Csn2*$^{WT/K70E}$ (Het) mice.** **(a)** Daily food intake of wildtype and Het mice (n = 4). Data are presented as Mean ± SD. P values were calculated by two-tailed Student's t-test. **(b-c)** Serum levels of free fatty acids **(b)** and triglycerides **(c)** after 6-h fasting (n = 4, n.s.: not significant, student's T test). Data are presented as Mean ± SD. **(d)** Intraperitoneal insulin tolerance test (ITT) of wildtype and Het mice at 4 month age (n = 4, ***$p < 0.001$, calculated by two way repeated-measures ANOVA testing Genotype-x-Time effect). Data are presented as Mean ± SD. 100% blood glucose means 9.94 ± 1.3 mM for WT, and 8.3 ± 1.1 mM for Het. **(e)** Effect of metformin (Met) treatment on insulin tolerance test of wildtype and *Csn2*$^{WT/K70E}$ (Het) mice (n = 5, ***$p < 0.001$, calculated by two way repeated-measures ANOVA testing Treatment-x-Time effect). Data are presented as Mean ± SD. 4-month old mice were treated with/without 250 mg/kg Metformin in drinking water for three weeks. 100% blood glucose means 8.8 ± 0.7 mM for WT without Metformin treatment, 8.4 ± 0.8 mM for WT with Metformin treatment, 9.2 ± 0.9 mM for Het without Metformin treatment, and 7.6 ± 0.9 mM for Het with Metformin treatment. **(f)** Effect of metformin (Met) treatment on serum insulin levels of wildtype and *Csn2*$^{WT/K70E}$ (Het) mice (n = 5). Data are presented as Mean ± SEM. P values were calculated by two-tailed Student's t-test. **(g)** Effect of streptozocin (STZ) treatment (i.p., 50 mg/kg for four days) on the body weight of wildtype and *Csn2*$^{WT/K70E}$ (Het) mice (n = 4, n.s.: not significant, calculated by two way ANOVA testing genotype main effect). Data are presented as Mean ± SEM.

## Supplementary Figure 2

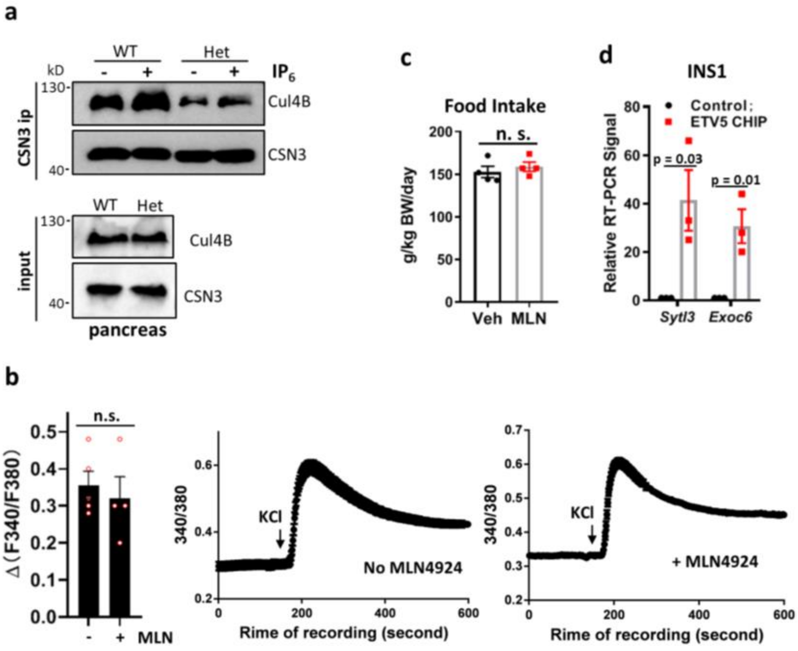


**Supplementary Fig. 2. Activation of the CRL4$^{COP1}$-ETV5 degradation axis underlies glucose-induced insulin secretion and is constitutively augmented in *Csn2$^{WT/K70E}$* (Het) mice. (a)** Effect of $IP_6$ (20 μM) addition to pancreatic lysate on CSN3-Cul4B co-immunoprecipitation from wildtype and *Csn2$^{WT/K70E}$* pancreas. **(b)** Effect of MLN4924 on average islet cytoplasmic $Ca^{2+}$ responses to depolarization induced by 25mM KCl, assayed with Fura2AM [n = 4 (WT), or 5 (Het), n.s.: not significant, student's T test]. Data are presented as Mean ± SEM. **(c)** Daily food intake of mice is not affected by MLN4924 treatment (n = 4, n.s.: not significant, student's T test). Data are presented as Mean ± SEM. P values were calculated by two-tailed Student's t-test. **(d)** ETV5 ChIP on Sytl3 and Exoc6 promoters (n = 3). Data are presented as Mean ± SEM. P values were calculated by two-tailed Student's t-test.

## Supplementary Figure 3

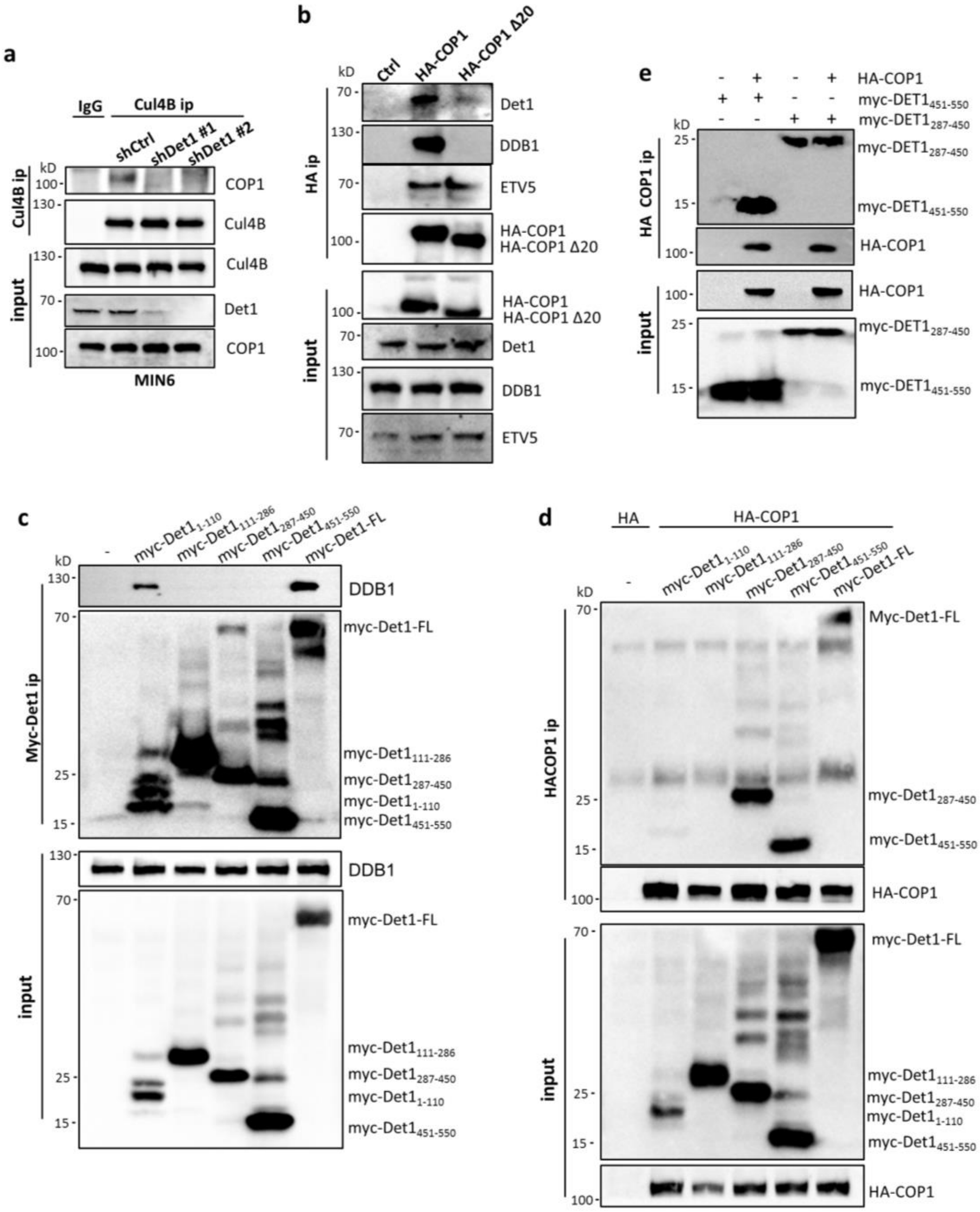


**Supplementary Fig. 3. Interaction domain characterization within the CRL4$^{COP1}$ complex. (a)** DET1 knockdown abolishes Cul4B immunoprecipitation of COP1. **(b)** Deleting COP1 aa 277-296 (Δ20) abolishes COP1 interaction with DET1 and DDB1, but not ETV5. **(c)** DET1 Fragment mapping experiments showing that DET1 aa 1-110 binds to DDB1. **(d-e)** Reciprocal co-immunoprecipiation experiments showing that DET1 aa 451-500 specifically interacts with COP1.

**Supplementary Figure 4**

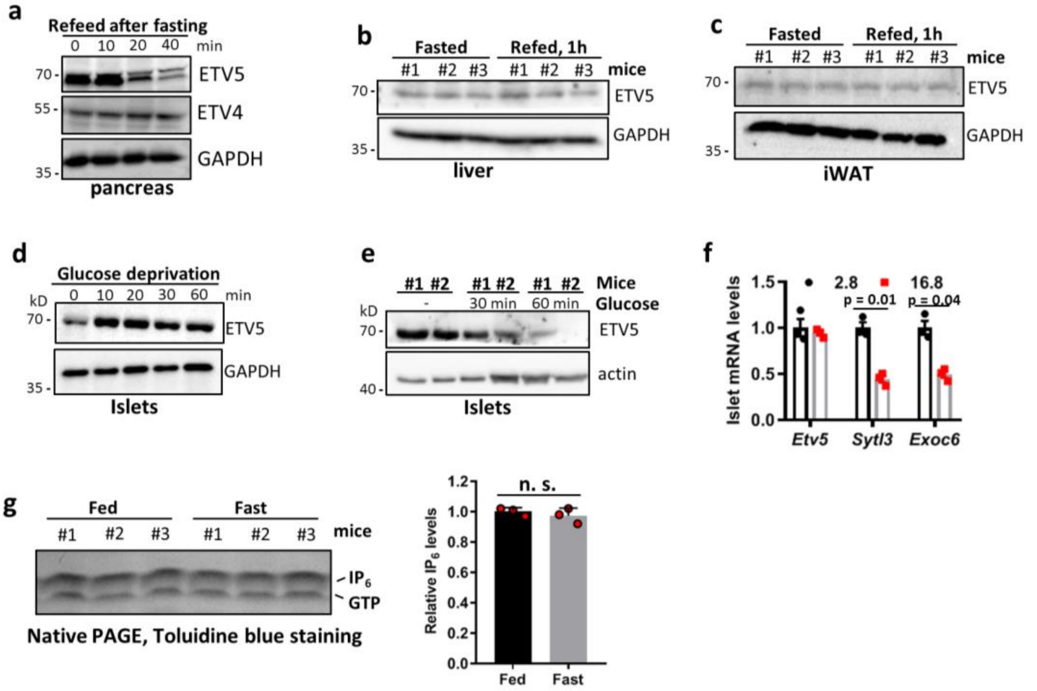


**Supplementary Fig. 4. ETV5 as a CRL4$^{COP1}$ regulated physiologic checkpoint for nutrient/glucose-induced insulin secretion. (a)** Levels of ETV5 in the pancreas of overnight-fasted mice, with/without refeeding for the indicated time periods. **(b-c)** Refeeding does not significantly change Levels of ETV5 in the liver **(b)** and adipose tissue **(c)** of fasted mice**. (d)** Levels of ETV5 in isolated islets cultured in RPMI mediated containing 11 mM glucose, followed by glucose withdrawal at the indicated time points. **(e)** Levels of ETV5 in isolated islets after exposure to 16.8 mM glucose at the indicated time points. **(f)** Transcript levels of Etv5 and its targets in islets incubated with 2.8 mM or 11.8 mM glucose (n = 3). Data are presented as Mean ± SEM. P values were calculated by two-tailed Student's t-test. **(g)** Total levels of cellular $IP_6$ in the pancreas of mice with/without fasting (6h) (n = 3, n.s.: not significant, student's T test). Data are presented as Mean ± SD.

# Supplementary Figure 5

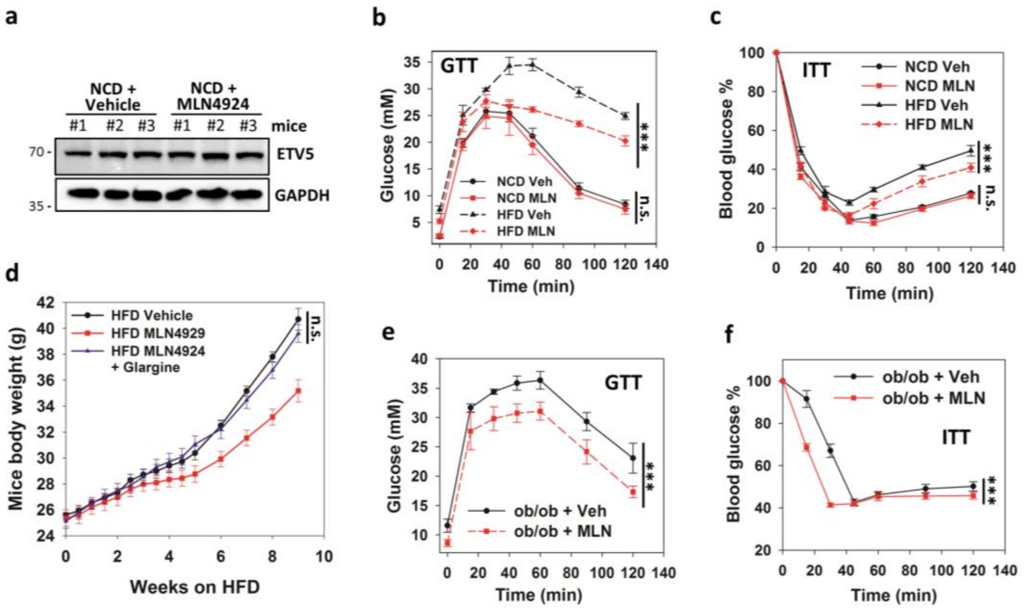


**Supplementary Fig. 5. The CRL inhibitor Pevonedistat/MLN4924 stabilizes ETV5 and improves diet-induced or leptin deficiency-induced obesity/diabetes**. **(a)** Effect of MLN4924 injection (i.p. 15 mg/kg) on ETV5 levels from mice fed with NCD. **(b-c)** Glucose **(b)** and insulin **(c)** tolerance test in HFD or NCD mice with/without MLN4924 treatment (n = 5, ***$p < 0.001$, calculated by two way repeated-measures ANOVA testing Treatment-x-Time effect). 100% blood glucose means 16.3 ± 2.2 mM for WT and 12.2 ± 1.4 mM for Het. 100% blood glucose means 7.2 ± 0.6 mM for NCD without MLN4924, 7.4 ± 0.5 mM for HFD with MLN4924, 9.8 ± 1.4 mM for HFD without MLN4924, and 7.6 ± 0.5 mM for HFD with MLN4924. **(d)** Concurrent treatment with Glargine (Sanofi, 50 IU/Kg, twice weekly), a slow-releasing insulin, reverses the anti-obese effect of MLN4924 (n = 5, n.s.: not significant, two way repeated-measures ANOVA testing Treatment-x-Time effect). Data are presented as Mean ± SD. Note that **(e-f)** Glucose **(e)** and insulin **(f)** tolerance test in ob/ob mice with/without MLN4924 treatment (n = 5, ***$p < 0.001$, calculated by two way repeated-measures ANOVA testing Treatment-x-Time effect). Data are presented as Mean ± SEM. 100% blood glucose means 16.3 ± 2.2 mM for ob/ob mice without MLN4924 treatment and 12.2 ± 1.4 mM for ob/ob mice with MLN4924 treatment.

**Supplementary Table 1. Primer information**

| Primers | Primer sequences |
|---|---|
| **A. Primers to generate different constructs** | |
| hCOP1-shRNA1 | GCTAACAGTCAGGGTACAATT |
| hCOP1-shRNA2 | GCTGGAGTTACAAAGAAGATT |
| hETV5-shRNA1 | GAGCGATACGTCTACAAATTT |
| hETV5-shRNA2 | TCCATGGCTTTCCCGGATAAC |
| mCOP1-shRNA1 | GCTGTATCAGTTGGAGTAGTT |
| mCOP1-shRNA2 | CCTTGGTATAACAGCACATTA |
| mDet1-shRNA1 | ATCAAGGGCTCTACTTGTATA |
| mDet1-shRNA2 | TCCTTGTTTCTTACGGAAATT |
| mEtv5-shRNA1 | GCCAAAGATGATGCCTGAA |
| mEtv5-shRNA2 | GCCATGAAGGATTCCCGTA |
| hSytl3-ECOR1-F | CTCGAGCTCAAGCTTCGAATTCATGGCCCAAGAAATAGATC |
| hSytl3-BamH1-R | CGTCGTCGTCCTTGTAGTCGGATCCTCAGTGCAGGACAAGAGTC |
| hExoc6-ECOR1-F | CTCGAGCTCAAGCTTCGAATTCATGGCGGAGAACAGCGAGA |
| hExoc6-BamH1-R | CGTCGTCGTCCTTGTAGTCGGATCCCTACATGTGCTGGGACATA |
| hDET1-F | AAAAGTCGACcATGGATCATCATGTTTCTACCA |
| hDET1-R | AAAAGCGGCCGCCTACGTGCAGCAGTGTCGCATAT |
| hDet1-1-110-F | GCGGAATTCGGTCGACCATGGAT |
| hDet1-1-110-R | GCGGCGGCCGCCTAGTCATTGCCATTGGACAGGA |
| hDet1-111-286-F | GCGGAATTCGGTCGACCCAGCGGTCAGTGAATATCC |
| hDet1-111-286-R | GCGGCGGCCGCCTAGGGATTGGCCATGCCT |
| hDet1-287-450-F | GCGGAATTCGGTCGACCTTTAGGGATCCTTTCATCAATTCC |
| hDet1-287-450-R | GCGGCGGCCGCCTAGATGGGGAGCTGACCC |
| hDet1-451-550-F | GCGGAATTCGGTCGACCAGTGCTCAGTCTTACAGCG |
| Det1-451-550-R | GCGGCGGCCGCCTACGTG |
| mDcaf8-shRNA | GCCATACTGGTTGTGTCAATA |
| mWdr59-shRNA | CTCCCTAGTGATTGCCTTCTT |
| mWdr51-shRNA | CCAGGTCTAATTGCAGTAATA |
| mWdr61-shRNA | GATGCCTGGACTTTGGCATTT |
| mWdr57-shRNA | GCAGGATTTGACTAATA |
| mDcaf5-shRNA | GAGGAAATGATGAGCAAGTTA |
| mDcaf9-shRNA | CTTACAAGCAACGGCCATATA |
| mDcaf14-shRNA | GGAGTCAAAGTTCGATCTTAT |
| mDcaf16-shRNA | CTCTAAATGGAGCACTGCAAT |

| | |
|---|---|
| mDcaf10-shRNA | TGATACCACAATAGCACTATG |
| mDcaf2-shRNA | TGATGAAGCTGCCTACATTTG |
| mDcaf11-shRNA | GAGCGGCCACATTGTTAAGAA |
| mDcaf4-shRNA | CTTCTCCAGTTATTGCCGTTT |
| mDcaf7-shRNA | CAACAACAAGAACTCAGACTT |
| mDcaf19-shRNA | GCAGCATATTTATATGGGATA |
| mDcaf1-shRNA | TGCAATTGGAAGACCTATTAT |
| mWdr12-shRNA | CCTACAGATGAAGAAGATGAA |
| mDcaf13-shRNA | CCCTGTTGAGACATTTCTCTT |
| mWdr39-shRNA | GCTGGAAATGTATCTGCACTT |
| mFbxw5-shRNA | GCGGCTCTTTAAGATCCAGAA |
| mRbbp4-shRNA | ATTTGGGACACTCGTTCAAAC |
| mWsb2-shRNA | CAATGGTCTTTGCTGCACGTT |
| mTtrpc4ap-shRNA | CTGATGAAGTTCAACGTCGAT |
| mEed-shRNA | TCTTGCTAGTAAGGGCACATA |
| mDdb2-shRNA | ATACCCAGATCCTAATCTTAA |
| mCsa-shRNA | GAGTTAAACAAAGACAGGGAT |
| mSMU1-shRNA | CCCATGATTATGTTAAAGCAA |
| mWsb1-shRNA | ACATGAGCTGCTGCTATATAT |
| mRbbp7-shRNA | CCTTTGATTCAACTGTCATTT |
| mDda1-shRNA | CTGCACCAGCAATGGGATAAA |
| mCrbn-shRNA | CCTACCAAGTTCAAGAGCATA |
| mDcaf17-shRNA | GAATCTGACTGGATCTATTTC |
| mWdr75-shRNA | CGAAGTCACAGAGCTTATTAA |
| **B: RT-PCR Primers** | |
| rSytl3-F | ATCCTGCACTGAGGTGAAGC |
| rSytl3-R | AGGCTCCCCGACAGTAATCT |
| rExoc6-F | AGATCGAAAGCACGGACACC |
| rExoc6-R | TCTCCTTGTCATGGTTGCGG |
| hETV5-F | TAGGCTAAGTTGGGGAGGCT |
| hETV5-R | GGGTGGTTCCCAAGAGTAGC |
| rIns1-F | CCAAGTCCCGTCGTGAAGT |
| rIns1-R | GGTGCAGCACTGATCCACAA |
| rGapdh-F | ATGACTCTACCCACGGCAAG |
| rGapdh-R | GGAAGATGGTGATGGGTTTC |
| mIns1-F | CACTTCCTACCCCTGCTGG |
| mIns1-R | ACCACAAAGATGCTGTTTGACA |
| mIns2-F | GCTTCTTCTACACACCCATGTC |
| mIns2-R | AGCACTGATCTACAATGCCAC |
| mSytl3-F | AGCTCCTGCAATCTTATCAGAGG |
| mSytl3-R | GCCAAGTTCCTGTAACCTGC |

| | |
|---|---|
| mExoc6-F | GAAAAGCTAGACGCTTGTATCCG |
| m Exoc6-R | TGAGGAGTTCCGTAATCGCAT |
| mGapdh-F | TCAAGAAGGTGGTGAAGCAG |
| mGapdh-R | AGGTGGAAGAGTGGGAGTTG |
