# Supplementary material for: IP6-assisted CSN-COP1 competition regulates a CRL4-ETV5 proteolytic checkpoint to safeguard glucose-induced insulin secretion
Source: Nat Commun. 2021 Apr 28;12:2461. doi: 10.1038/s41467-021-22941-3 (PMC8080631; doi:10.1038/s41467-021-22941-3)
Supplement: Supplementary file 3 — Reporting Summary [file 41467_2021_22941_MOESM3_ESM.pdf]

natureresearch

Corresponding author(s): Feng Rao

Last updated by author(s): Mar 12, 2021

Reporting Summary

Nature Research wishes to improve the reproducibility of the work that we publish. This form provides structure for consistency and transparency in reporting. For further information on Nature Research policies, see Authors & Referees and the Editorial Policy Checklist .

Statistics

For all statistical analyses, confirm that the following items are present in the figure legend, table legend, main text, or Methods section.

n/a	Confirmed	
☐	☒	The exact sample size (*n*) for each experimental group/condition, given as a discrete number and unit of measurement
☐	☒	A statement on whether measurements were taken from distinct samples or whether the same sample was measured repeatedly
☐	☒	The statistical test(s) used AND whether they are one- or two-sided<br>*Only common tests should be described solely by name; describe more complex techniques in the Methods section.*
☐	☒	A description of all covariates tested
☒	☐	A description of any assumptions or corrections, such as tests of normality and adjustment for multiple comparisons
☐	☒	A full description of the statistical parameters including central tendency (e.g. means) or other basic estimates (e.g. regression coefficient) AND variation (e.g. standard deviation) or associated estimates of uncertainty (e.g. confidence intervals)
☐	☒	For null hypothesis testing, the test statistic (e.g. *F*, *t*, *r*) with confidence intervals, effect sizes, degrees of freedom and *P* value noted<br>*Give P values as exact values whenever suitable.*
☒	☐	For Bayesian analysis, information on the choice of priors and Markov chain Monte Carlo settings
☒	☐	For hierarchical and complex designs, identification of the appropriate level for tests and full reporting of outcomes
☒	☐	Estimates of effect sizes (e.g. Cohen's *d*, Pearson's *r*), indicating how they were calculated

Our web collection on statistics for biologists contains articles on many of the points above.

Software and code

Policy information about availability of computer code

Data collection	The data for Figure 4A was generated using the online software I-Tasser v5.1, which is available at: https://zhanglab.ccmb.med.umich.edu/I-TASSER/. Other software used include: Metafluor v7.8.12.0 (Calcium imaging data collection); Synergy H1 (ELISA data collection); Excel 2010; Graphpad Prism v8.0.1; Image-Pro Plus software; Electron Microscope (Hitachi TEM-HT7700) .
Data analysis	Pymol2.2.0 (for structural analysis and figure generation); Graphpad Prism (for figure generation)

For manuscripts utilizing custom algorithms or software that are central to the research but not yet described in published literature, software must be made available to editors/reviewers. We strongly encourage code deposition in a community repository (e.g. GitHub). See the Nature Research guidelines for submitting code & software for further information.

Data

Policy information about availability of data

All manuscripts must include a data availability statement. This statement should provide the following information, where applicable:

- Accession codes, unique identifiers, or web links for publicly available datasets
- A list of figures that have associated raw data
- A description of any restrictions on data availability

The uniprot id for COP1 and DET1 proteins sequences are Q7L5Y6 and Q8NHY2, respectively. All data generated or analyzed during this study are included in this article and its supplementary information files, or are available from the corresponding author upon request. Data and reagents requests should be addressed to F. Rao (raof@sustech.edu.cn).

Field-specific reporting

Please select the one below that is the best fit for your research. If you are not sure, read the appropriate sections before making your selection.

- [x] Life sciences
- [ ] Behavioural & social sciences
- [ ] Ecological, evolutionary & environmental sciences

For a reference copy of the document with all sections, see nature.com/documents/nr-reporting-summary-flat.pdf

Life sciences study design

All studies must disclose on these points even when the disclosure is negative.

Sample size	For animal experiments, the number of mice represent the sample size. Sample size were empirically chosen during experimental design and is present in figure legends. No sample size calculation was performed. For other experiments, the number of sample refer to experimental replicates and are empirically chosen during experimental design. No sample size calculation was performed.
Data exclusions	No data were excluded from the analysis.
Replication	All western blot data are representative of two or more experimental replicates. Other data (calcium image, EM, fluorescence staining) are also replicated with the number of samples shown in the plot as the number of individual datapoints.
Randomization	In genetic analysis, mice were grouped according to genetic identity. In pharmacological studies, mice were randomly grouped. In other studies, there is no randomization, and experimental conditions were clearly described in figure labels.
Blinding	Investigators were blinded to group allocation during data collection and/or analysis.

Reporting for specific materials, systems and methods

We require information from authors about some types of materials, experimental systems and methods used in many studies. Here, indicate whether each material, system or method listed is relevant to your study. If you are not sure if a list item applies to your research, read the appropriate section before selecting a response.

Materials & experimental systems

n/a	Involved in the study	
[ ]	[x]	Antibodies
[ ]	[x]	Eukaryotic cell lines
[x]	[ ]	Palaeontology
[ ]	[x]	Animals and other organisms
[ ]	[x]	Human research participants
[x]	[ ]	Clinical data

Methods

n/a	Involved in the study	
[x]	[ ]	ChIP-seq
[x]	[ ]	Flow cytometry
[x]	[ ]	MRI-based neuroimaging

Antibodies

Antibodies used	Cul1 (1:1000, CST, 4995), Cul3 (1:1000, CST, 2759), CSN5 (1:1000, CST, 6895), CDT1 (1:1000, Proteintech, 14382-1-AP); ETV1 (1:1000, abcam, ab184120), ETV5 (1:1000, abcam, ab102010, CHIP and WB), Cul4A (1:1000, abcam, ab72548, WB and IP), CSN3 (1:1000, abcam, ab79698), Cul2 (1:1000, Bethyl, A302–476A), Cul5 (1:1000, Bethyl, A302–173A), COP1 (1:1000, Bethyl, A302–173A), CSB (1:1000, Genetex, GTX104589), GAPDH (1:3000, Proteintech, 10494-1-AP), Exoc6 (1:1000, Proteintech, 12723-1-AP), Sytl3 (1:1000, Proteintech, 22076-1-AP), CSN2 (1:3000, Proteintech, 10969-2-AP); ETV4 (1:1000, Thermo, MA5-15424); Cul4B (1:1000, Sigma, C9995, WB and IP), GST (1:3000, Sigma, A7340); Det1 (1:1000, Santa cruz, sc-514348); Insulin (1:100, R&D Systems, I2018, for IF), Glucagon (1:100, abcam, ab10988, for IF)
Validation	Anti-ETV5 (abcam, ab102010), anti-COP1 (Bethyl, A302–173A), and anti-Det1 (Santa cruz, sc-514348) were validated using knockdown cells as controls. Anti-Cul1 (CST, 4995), anti-Cul2 (Bethyl, A302–476A), anti-Cul3 (CST, 2759), anti-Cul4A (abcam, ab72548), anti-Cul4B (Sigma, C9995), anti-Cul5 (Bethyl, A302–173A), anti-CSN2 (Proteintech, 10969-2-AP), anti-GAPDH (Proteintech, 10494-1-AP), and anti-GST (Sigma, A7340) antibodies were validated in previous studies from our laboratory (PNAS, 2016, 113, 3503-; PNAS 2020,117, 4117-; JBC 2020, 295, 10281-). Anti-Insulin (R&D Systems, I2018), anti-Glucagon (abcam, ab10988), Anti-CDt1 (Proteintech, 14382-1-AP), anti-ETV1 (abcam, ab184120), anti-ETV4 (Thermo, MA5-15424), anti-CSN3 (abcam, ab79698), anti-CSB (Genetex, GTX104589), anti-Exoc6 (Proteintech, 12723-1-AP), anti-Sytl3 (Proteintech, 22076-1-AP), and anti-CSN5 (CST, 6895)were validated for detecting mouse proteins in the manufacturers' websites.

Eukaryotic cell lines

Policy information about cell lines

Cell line source(s)	HEK293, HEK293T, INS1 and MIN6 cells were purchased from ATCC. EndoC-bH1 cells were purchased from the company EndoCells (Paris, France).
Authentication	None of the cell lines used were authenticated.
Mycoplasma contamination	All cell lines tested negative for mycoplasma contamination.
Commonly misidentified lines (See ICLAC register)	No commonly misidentified cell line is used in this study.

Animals and other organisms

Policy information about studies involving animals; ARRIVE guidelines recommended for reporting animal research

Laboratory animals	Male C57BL6 mice were used. Mice age differs and are specific for a particular experiment, which could be an age-dependent experiment. Therefore, age was clearly indicated in figure legends and figure labels.
Wild animals	The study did not involve wild animals.
Field-collected samples	The study did not involve samples collected from the field.
Ethics oversight	All mice were maintained in specific pathogen-free (SPF) facilities, and experiments were conducted following the Southern University of Science and Technology Guide for the Care and Use of Laboratory Animals, and approved by the Animal Ethics Committee (Approval code: SUSTC-2017-076)

Note that full information on the approval of the study protocol must also be provided in the manuscript.

Human research participants

Policy information about studies involving human research participants

Population characteristics	Our study utilized pancreatic tissues from human donors with informed consent. Characteristics of the donors (e.g. age, gender, etc) were collected by dedicated Organ Procurement Organization in a double-blinded manner. As such, we have no access to these information. Nonetheless, the donors are metabolically healthy for transplantation surgery, as evaluated by the OPO.
Recruitment	Three available human islet prepartions were studied. Donors are metabolically healthy and are concurrently donating for transplantaiton surgery. There is no self-selection bias or other biases.
Ethics oversight	The Ethics Committee of the Third People's Hospital of Shenzhen

Note that full information on the approval of the study protocol must also be provided in the manuscript.
